# Isolation and complete genome sequence analysis of a novel ovine adenovirus type representing a possible new mastadenovirus species

**DOI:** 10.1007/s00705-019-04299-6

**Published:** 2019-05-31

**Authors:** Márton Z. Vidovszky, Levente Szeredi, Andor Doszpoly, Balázs Harrach, Ákos Hornyák

**Affiliations:** 1 Institute for Veterinary Medical Research, Centre for Agricultural Research, Hungarian Academy of Sciences, P.O. Box 18, 1143 Budapest, Hungary; 2 Veterinary Diagnostic Directorate, National Food Chain Safety Office, 1143 Budapest, Hungary

## Abstract

Pathological examination of a suckling male lamb showed severe viral pneumonia with suspected bacterial superinfection. Adenovirus was detected by immunohistochemical examination of the affected lung samples. Detection of the suspected adenovirus by PCR and subsequent isolation of the virus were successful. Using next-generation sequencing, the full genome of this ovine adenovirus was sequenced and analysed. A genome sequence comparison showed that it was a novel mastadenovirus type (named “ovine adenovirus 8”) that did not belong to any of the established adenovirus species. The genome is 36,206 bp long, containing 93-bp inverted terminal repeats and 29 predicted genes, including the two genus-specific genes (encoding proteins V and IX). Ovine adenovirus 8 shows the closest relationship to ovine adenovirus 6. These two viruses seem to merit the establishment of a novel ovine mastadenovirus species for them, for which we proposed the name “*Ovine mastadenovirus C*”.

**Electronic supplementary material:**

The online version of this article (10.1007/s00705-019-04299-6) contains supplementary material, which is available to authorized users.

Adenoviruses (AdVs) are medium-sized, non-enveloped viruses with linear dsDNA genomes. AdVs have been reported in almost all known classes of vertebrates (mammals, birds, reptiles, amphibians and fish) [1–7]. Phylogenetic analysis has shown that most AdVs infecting mammals belong to the genus *Mastadenovirus*. Ruminants are the only mammals that have AdVs belonging to the genus *Atadenovirus* as well. So far, seven types of AdVs from sheep have been described [1], belonging to three distinct AdV species accepted by the International Committee on Taxonomy of Viruses (ICTV) [3]. Two species, *Ovine mastadenovirus A* and *Ovine mastadenovirus B* belong to the genus *Mastadenovirus*. Ovine adenoviruses 2-5 (OAdV-2-5) and bovine adenovirus 2 (BAdV-2) belong to *Ovine mastadenovirus A*, while OAdV-1 and caprine adenovirus 2 (from goat, GAdV-2) belong to *Ovine mastadenovirus B*. OAdV-7 and GAdV-1 belong to the species *Ovine atadenovirus D* of the genus *Atadenovirus*. Only partial genome sequences of the known OAdVs belonging to the genus *Mastadenovirus*, mostly from the hexon gene, are available in the GenBank database [6]. The only fully sequenced OAdV genome is that of the OAdV-7 [12]. There has not been a reported isolation or even a PCR detection of a novel type of AdV in sheep since 1983.

In 2017, a pathological and histopathological examination of a suckling male lamb showed severe viral pneumonia characteristic of adenoviral infection with suspected bacterial superinfection in the Veterinary Diagnostic Directorate, National Food Chain Safety Office of Hungary. Immunohistochemical examination (IHC) demonstrated the presence of adenovirus in the cytoplasm of few enlarged inclusion-bearing bronchiolar and alveolar epithelial cells (Fig. 1). A novel type of OAdV (strain 7508) was isolated and propagated in the OA3 (ovine testicle) cell line. The virions were concentrated by ultracentrifugation, and the viral DNA was purified using the phenol/chloroform extraction method. Next-generation sequencing (NGS) was performed on an Illumina MiSeq platform. A total of 13,073,060 reads were generated, with an average length of 70 bp. CLC Genomics Workbench 8.5 (CLC bio) was used for sequence assembly. Two contigs were obtained, and the gap between them was filled by PCR amplification. To determine the ends of the genome, we used direct sequencing with specific primers. The programs used for handling, identification and analysis of nucleotide sequences have been described in detail elsewhere [4]. The complete genome sequence of the novel OAdV (named “ovine adenovirus 8” [OAdV-8]) was deposited in the GenBank database under accession no. MK518392.

**Fig. 1:**
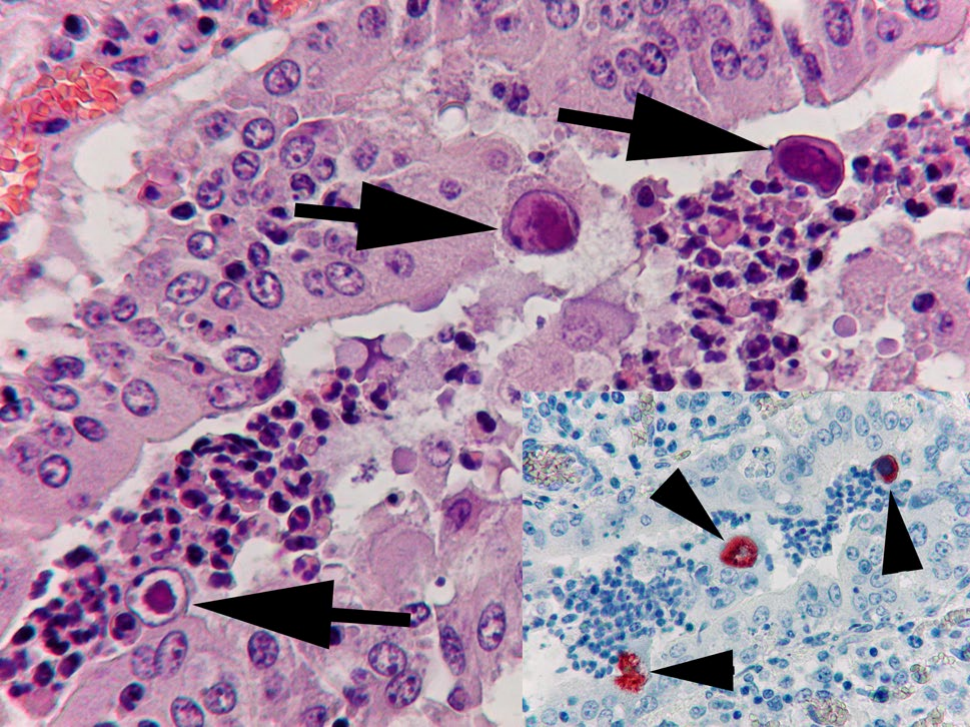
Enlarged bronchiolar epithelial cells containing intranuclear inclusion bodies (arrows), and acute purulent bronchiolitis in a bronchiole (haematoxylin and eosin staining). Inset: Immunostaining of the cytoplasm of enlarged bronchiolar epithelial cells (arrowheads), showing the presence of adenoviral antigen (IHC)

Here, we report the first complete genome sequence of an OAdV belonging to the genus *Mastadenovirus*. The genome of OAdV-8 is 36,206 bp long, and the inverted terminal repeats (ITRs) at the genome ends are 93 bp long. The G+C content of the whole genome proved to be unexpectedly high (70%). OAdV-8 shows a typical mastadenovirus genome organization, containing the genus-specific genes encoding proteins V and IX and the E1, E3 and E4 regions. Twenty-nine genes were predicted in the OAdV-8 genome (Fig. 2), all of them showing clear homology to the genes of mastadenoviruses. During the annotation, all of the expected splicing sites were identified in the genes for E1A, IVa2, DNA-dependent DNA polymerase, pTP, and 33K. The gene for the whole U exon protein (UXP) was also identified by predicting splicing sites for three exons. UXP was first detected in the genome of human adenovirus 5 [10] but was later predicted also in other primate (Old World monkey) AdVs [8]. The less-conserved E4 region contains four ORFs, each showing sequence similarity (43-57% amino acid [aa] sequence identity) to the corresponding ORFs of the E4 region of bovine adenovirus 3 (BAdV-3) and deer AdV-2 [9]. The E3 region of mastadenoviruses usually contains several ORFs, and in the E3 region of OAdV-8, we identified a homologue of the BAdV-3 and deer AdV-2 E3 ORFA. This short E3 region is quite unique in ruminant AdVs, since all known E3 regions of mastadenovirus BAdVs except that of BAdV-10 are longer [11]. Nevertheless, the BAdV-3 E3 region encodes a second protein (E3 14.7K). Neither OAdV-8 nor BAdV-3 contained the E3 12.5K gene, which is present in almost every mastadenovirus [2]. On the other hand, E3 regions have not yet been sequenced from any other mastadenovirus OAdVs. This short E3 region is more typical of some rodent and bat AdVs [4, 5].

**Fig. 2:**
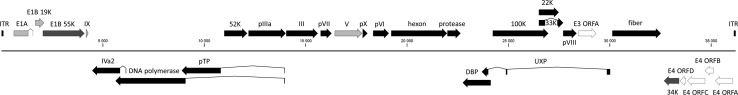
Genome map of ovine adenovirus 8. A grey scale is used to indicate genes that have homologs in other adenoviruses. Black, genes present in all AdVs; dark grey, genes occurring in both mastadenoviruses and atadenoviruses; light grey, genes specific to mastadenoviruses; white, genes with homologs only in bovine adenovirus 3

Sequence analysis showed that the closest relative of OAdV-8 is OAdV-6. This serotype has not been assigned to any existing ovine mastadenovirus species. For OAdV-6, only the hexon gene sequence is available in the GenBank database. This region shows 95% aa sequence identity to the corresponding region of OAdV-8. However, when only the first hexon loop sequences of the two viruses are compared, the aa sequence identity drops to 82%. This part of the hexon is responsible for antigenicity, suggesting that the two viruses are distinct serotypes. Meanwhile, the high sequence similarity of the entire hexon gene implies that the two viruses may belong to the same virus species. Since the demarcation criterion for AdV species classification by the ICTV is based on the DNA-dependent DNA polymerase gene, it would be necessary at least to determine the sequence of the polymerase gene from OAdV-6 to confirm this prediction. Regardless of that status of OAdV-6, providing the complete genome sequence of a novel, distinct type of ovine mastadenovirus, we propose to designate OAdV-8 as a founding member of a novel mastadenovirus species (with the proposed name “*Ovine mastadenovirus C*”) based on the following demarcation criteria: 36% difference in the DNA polymerase protein sequence from the most similar AdV, a member of another species (BAdV-3, species *Bovine mastadenovirus B*; demarcation criterion, 5–15% difference), host, (sheep vs. cattle or deer), GC percentage (70% vs. 54% for BAdV-3), and gene content differences in the E3 region. The high GC content seems to suggest that OAdV-8 represents the original lineage that coevolved with members of the order Artiodactyla.

## Electronic supplementary material

Below is the link to the electronic supplementary material.

Supplementary material 1 (GBK 68 kb)
